# Transcriptomic analysis of *Perilla frutescens* seed to insight into the biosynthesis and metabolic of unsaturated fatty acids

**DOI:** 10.1186/s12864-018-4595-z

**Published:** 2018-03-21

**Authors:** BingNan Liao, YouJin Hao, JunXing Lu, HuiYang Bai, Li Guan, Tao Zhang

**Affiliations:** Collage of Life Sciences, Chongqing Normal University, Chongqing, 401331 China

**Keywords:** *Perilla frutescens*, Fatty acid biosynthesis, RNA-seq, Seed development, Gene expression profiling

## Abstract

**Background:**

*Perilla frutescens* is well known for its high α-linolenic acid (ALA) accumulation in seeds and medicinal values as well as a source of edible and general-purpose oils. However, the regulatory mechanisms of the biosynthesis of fatty acid in its seeds remain poorly understood due to the lacking of sequenced genome. For better understanding the regulation of lipid metabolism and further increase its oil content or modify oil composition, time-course transcriptome and lipid composition analyses were performed.

**Results:**

Analysis of fatty acid content and composition showed that the α-linolenic acid and oleic acid accumulated rapidly from 5 DAF to 15 DAF and then kept relatively stable. However, the amount of palmitic acid and linoleic acid decreased quickly from 5 DAF to 15DAF. No significant variation of stearic acid content was observed from 5 DAF to 25DAF. Our transcriptome data analyses revealed that 110,176 unigenes were generated from six seed libraries at 5, 10, 20 DAF. Of these, 53 (31 up, 22 down) and 653 (259 up, 394 down) genes showed temporal and differentially expression during the seed development in 5 DAF vs 10 DAF, 20 vs 10 DAF, respectively. The differentially expressed genes were annotated and found to be involved in distinct functional categories and metabolic pathways. Deep mining of transcriptome data led to the identification of key genes involved in fatty acid and triacylglycerol biosynthesis and metabolism. Thirty seven members of transcription factor family *AP2*, *B3* and *NFYB* putatively involved in oil synthesis and deposition were differentially expressed during seed development. The results of qRT-PCR for selected genes showed a strong positive correlation with the expression abundance measured in RNA-seq analysis.

**Conclusions:**

The present study provides valuable genomic resources for characterizing *Perilla* seed gene expression at the transcriptional level and will extend our understanding of the complex molecular and cellular events of oil biosynthesis and accumulation in oilseed crops.

**Electronic supplementary material:**

The online version of this article (10.1186/s12864-018-4595-z) contains supplementary material, which is available to authorized users.

## Background

*Perilla* is a self-pollinating plant and widely distributed in East Asian countries and considered as a food supply and natural medicine resource [1, 2]. *Perilla frutescens* belongs to the Lamiaceae family and encompasses many natural varieties [3]. *P. frutescens* var. *frutescens* seed has potential application in pharmaceutical and food industry due to the high accumulation of unsaturated acids, such as α-linolenic acid (ALA, 18:3) (> 60% of total FA in seeds) [4, 5]. With the exception of flaxseed oil, such high levels of ALA are uncommon in seed oils. However, the variety *P. frutescens* var. *crispa* has been used as a Chinese medicine or spicy vegetable crop [6, 7].

ALA content in most common edible oils, including peanut oil, sesame oil, sunflower oil and olive oil, is less than 3% [8, 9] . High ALA content in *Perilla* seeds, ranging from 60 to 70% depending on the varieties, is not only beneficial to human health but also important for stress responses, pathogen defense-related signaling and cell maturation processes. Therefore, it is a good model plant to dissect the biosynthesis pathways of unsaturated fatty acids. Although, few genes encoding enzymes involved in fatty acid biosynthesis, such as FAD3, 3-ketoacyl-ACP synthases, KAS (I, II, and III), have been characterized in *Perilla* [10, 11], the molecular regulatory mechanisms underlying the biosynthesis and metabolism of FA in *Perilla* seed have not yet been intensively studied, largely due to a dearth of genetic resources. To insight into the conserved and diverse aspects of lipid metabolism across multiple species, it is useful to expand the genomic and transcriptomic datasets available for non-model species to facilitate comparative analyses. Without the genome sequence of *Perilla*, transcriptome sequencing is an effective approach to identify the genes involved in specific biological processes.

As an initial step to understand the expression patterns of genes associated with fatty acid biosynthesis in *Perilla*, an cDNA library of *P. fruescens* [12] was constructed and 1056 expressed sequence tags were identified [13]. Subsequently, comparative expression profiles within cultivar variety *P. frutescens* var. *frutescens* Britt and wildtype *P. frutescens* var. *crispa* were performed through the de novo transcriptome sequencing approach, candidate genes causing the different leaf color and seed size were identified and their expression patterns were compared [14]. These datasets provided a large number of targeted gene information and could be referenced for functional transcriptome studies of *Perilla*. However, due to the temporal and spatial characteristics of transcriptome, from these studies it is not clear how fatty acid and TAG biosynthesis pathways were regulated and how they affect the oil content, composition and accumulation during seed development. Therefore, it is necessary to explore the transcriptome of developing seeds for further understanding of the regulation of lipid metabolism. To accomplish this objective, FA content and compositions in developing seeds were analyzed and a time-series analysis of transcriptomic data was performed. The approach used here is helpful to systematically identify the core biological process involved in oil synthesis and the new identified transcription factors will help us to further explore the molecular regulatory mechanisms of oil biosynthesis and metabolism in developing seeds.

## Results and discussion

### Major fatty acid content and composition differ from developing seeds

**Fig. 1:**
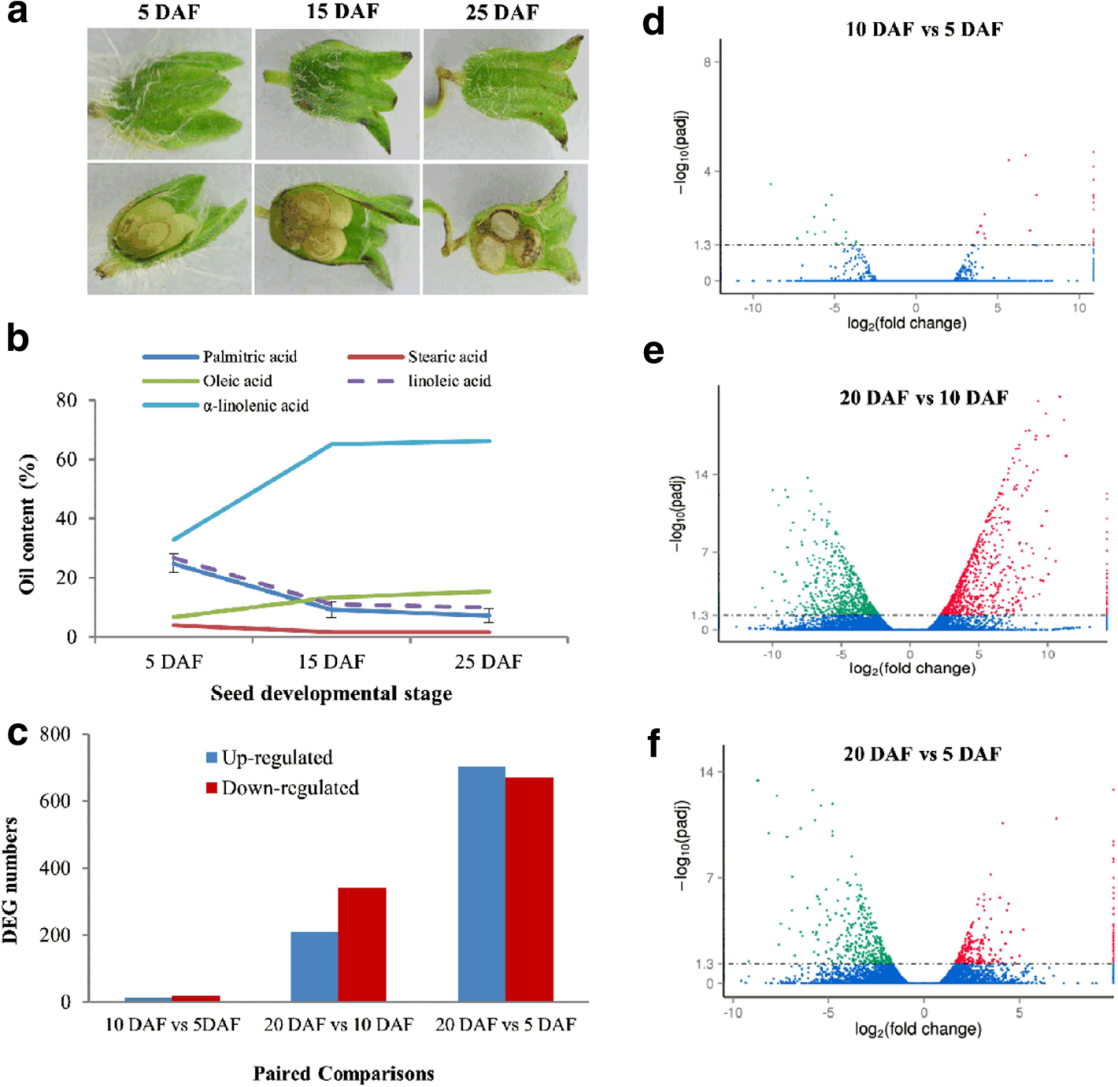
Seed development stages, lipid content and composition, and differentially expressed genes analysis during seed development. **a**: The three developing stage after flowing used for lipid content and composition analysis; **b**: lipid content and composition; **c**: Differentially expressed genes in three paired comparisons (10 DAF vs 5 DAF, 20 DAF vs 10 DAF, and 20 DAF vs 5 DAF); **d-f**: Distribution of differentially expressed genes based on log2 FC values in three paired comparisons

To obtain time-series of oil content variation during seed development, *Perilla* seeds at 5, 15 and 25 DAF were selected for oil content and composition measurements. The images of bud and dissection of seed from these three development stages were shown in Fig. 1a. Five dominant components were observed in developing seeds, including palmitic acid (C16:0), stearic acid (C18:0), oleic acid (C18:1), linoleic acid (C18:2) and α-linolenic acid (C18:3) (Fig. 1b). The content of palmitic acid (19.43%) and stearic acid (5.20%) was higher in 5 DAF seeds than in 15 DAF or 25 DAF seeds. In developing seeds, a relatively stable proportion of oleic acid (16.98% for 5 DAF, 13.24% for 15 DAF and 13.22% for 25 DAF) was observed. However, the content of linolenic acid decreased dramatically from 26.81% in 5 DAF seeds to 10.87% in 15 DAF seeds, and then remained relatively steady until 25 DAF (14.25%). On the contrary, the content of α-linolenic acid increased notably from 26.95% in 5 DAF seeds to 65.10% in 15 DAF seeds, and maintained a stable level in 25 DAF seeds (66.15%). Overall, the content variation of major compositions in mid-developing stage (15 DAF) and late-developing stage (25 DAF) was not significant, which provides a platform to elucidate how fatty acid biosynthesized in *Perilla* seeds.

### Transcriptome sequencing and de novo assembly

To gain a comprehensive transcriptional profile of *Perilla* seeds, sample collecting at the optimal developmental stage is crucial. It was rationalized that gene expressions and their regulations would precede the presence of enzymes and their products. Therefore, seeds at 5 DAF, 10 DAF and 20 DAF were chosen to explore the regulation of fatty acid biosynthesis and metabolism. Two cDNA libraries for each developmental stage were constructed and sequenced by the Illumina sequencing technology. Around 60 million RNA-seq reads were obtained for each developmental stage (Additional file 1:Table S1). After stringent quality control and data filtering, about 57.8 M, 55.7 M, and 57.6 M clean reads were generated for 5 DAF, 10 DAF and 20 DAF, respectively. A total of 25.53G data were further de novo assembled into 110,176 unigenes with an average length of 758 bp and an N50 of 1420 bp. Among of these unigenes, 22,492 unigenes were longer than 1 kb and account for 20.4% of the total unigenes. The length distribution of all unigenes was shown in Additional files 2 and 3: Figure S1 A and Table S2. These results revealed that the assembled data were qualified for further analyses.

### Functional annotations of unigenes

For gene functional annotation, all assembled unigenes were aligned by BLAST search against the NR, NT, KO, SwissProt, Pfam, GO and KOG databases (e-value< e-5), which retrieved proteins with the highest sequences identities with the given unigene along with their functional annotations. Blast search showed 47.07% unigenes had significant match to genes in the NR database, followed by 37.51% in Swissport database, 34.66% in GO database, 34.37% in Pfam database, 26.10% in NT database, 21.16% in KOG database and 16.42% in KO database (Additional files 2 and 3: Figure S1 B and C, Table S2). The remaining unmatched unigenes could be attributable to the short sequence reads generated by the sequencing technology, or might be unique to *P. frutescens*, or the relatively short sequences lacked conserved functional domains.

To identify the species specificity, individual unigene was annotated based on the highest BLAST score against the NR database. Among higher plants, 39.8% unigenes had close homology to *Sesamum indicum*, then followed by *Erythranthe guttata* (10.4%), *Brassica napus* (5.9%), *Hordeum vulgare* (1.7%) and *Vitis vinifera* (1.7%) (Additional file 2: Figure S1 D). Further analysis indicated that 51.6% unigenes of the top hits showed very strong homology (E-value < 1.0e^− 45^), while 48.4% of the matched unigenes showed moderate homology with an E-value between 1.0e^− 5^ and 1.0e^− 45^ (Additional file 2: Figure S1 E). The similarity analysis showed that 51.2% unigenes had a similarity higher than 80%, 48.7% unigenes shared 40–80% similarity, and 0.1% unigenes were lower than 40% similarity (Additional file 2: Figure S1 F).

**Fig. 2:**
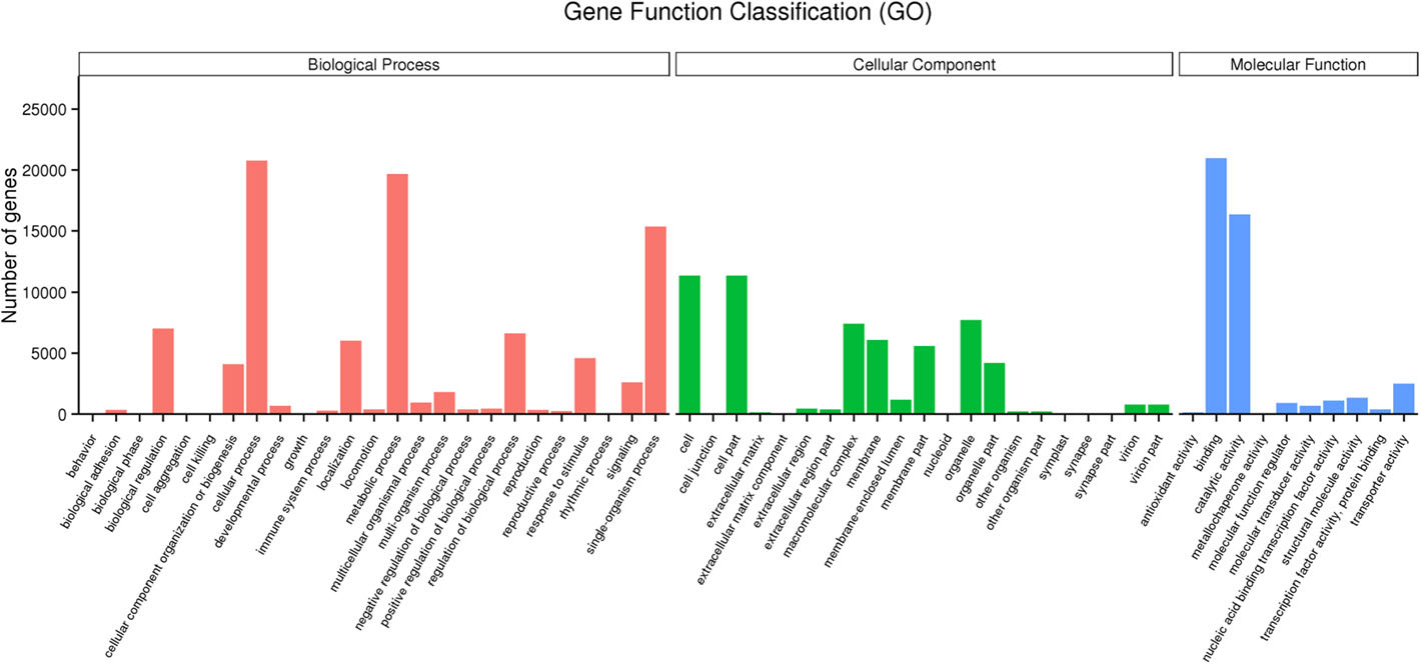
Gene ontology categories of all assembled unigenes. Unigenes were assigned into three main categories: biological processes, cellular components or molecular functions. The y-axis indicates the number of unigenes in a given category

Based on the *Arabidopsis* Information Resource Gene Ontology Slim classification system, 38,195 unigenes were categorized into 56 functional groups (Fig. 2). In the biological process category, “cellular process” (26.97%) was the most represented GO term, followed by “metabolic process” (16.96%), “single-organism process” (9.67%), “biological regulation” (7.75%), “localization” (6.64%) and “regulation of biological process” (6.01%). Within the cellular component category, the great majority unigenes were related to “cell” (31.38%) and “cell part” (24.80%), followed by “macromolecular complex” (17.27%), “organelle” (12.42%), and “membrane” (9.68%). With regard to molecular function category, the predominant categories were “binding” (other binding, 39.97%) and “catalytic activity” (22.17%), followed by “transporter activity” (9.09%), “structural molecule activity” (8.57%), “nucleic acid binding transcription factor activity” (8.06%) and “molecular function regulator” (7.96%). This distributions of genes in different categories in three *P. frutescens* seed developing stages were different from transcripts of leaves of red and green forms of the *P. frutescens* var. *crispa* [15, 16] due to the spatial and temporal characteristics of transcripts.

### KOG and KEGG classification

**Fig. 3:**
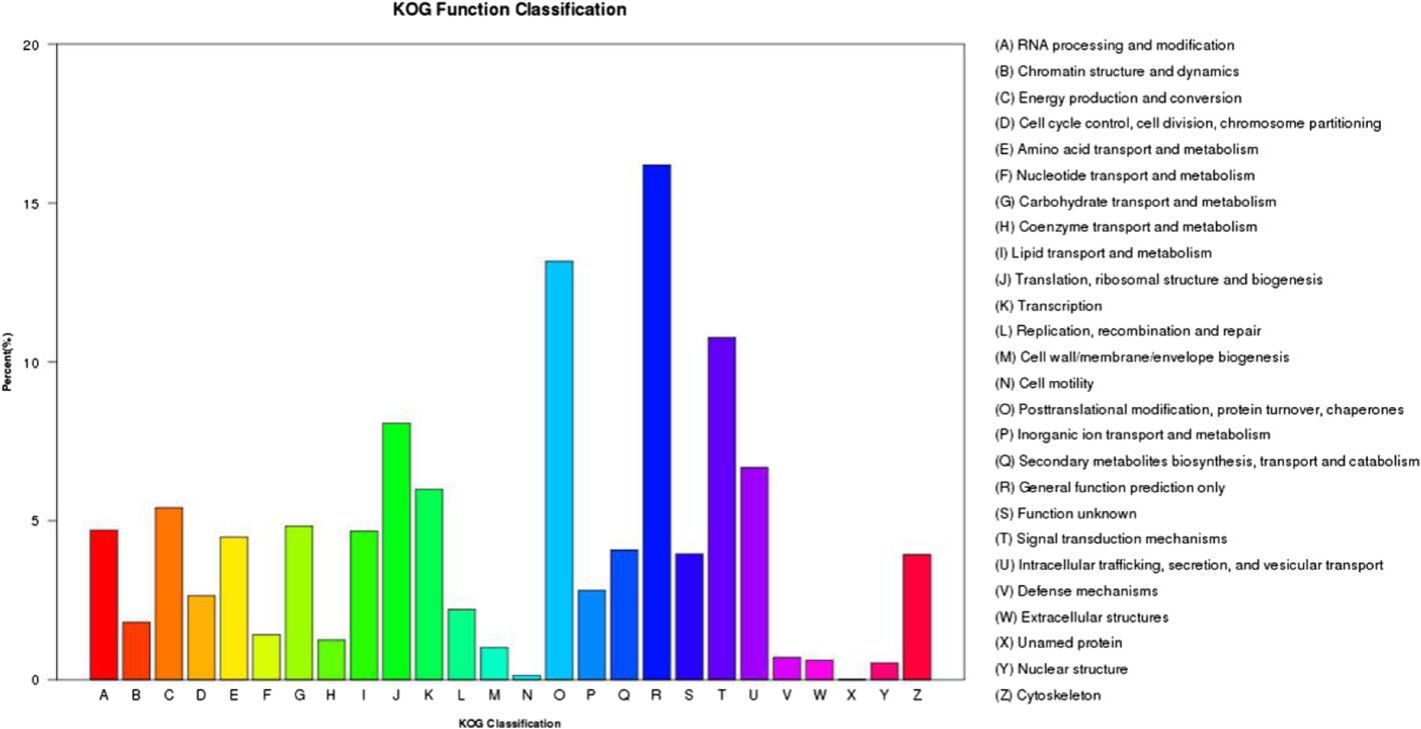
KOG functional classification of all unigenes. The unigenes were classified into different functional clusters based on KOG annotations

For functional prediction and classification, the annotated unigenes were mapped to the eukaryotic orthologous groups (KOG) database. In total, 23,316 annotated putative proteins (21.16%) were classified into 26 KOG groups (Fig. 3). Among these categories, a large number of transcripts were assigned to “general function prediction only” (3777; 16.19%), followed by the transcripts associated with “post-translational modification, protein turnover and chaperones” (3068; 13.16%);“signal transduction mechanisms” (2511; 10.76%); “translation, ribosomal structure and biogenesis” (1880, 8.06%). The category of “cell motility” (32; 0.4%), “nuclear structure” (124; 0.53%) and “extracellular structures” (143; 0.61%) represented the small groups. Notably, 1090 proteins (4.67%) were classified into “lipid transport and metabolism”, which we focus on and will provide useful data for further studies on lipid metabolism in *P. frutescens* seed.

**Fig. 4:**
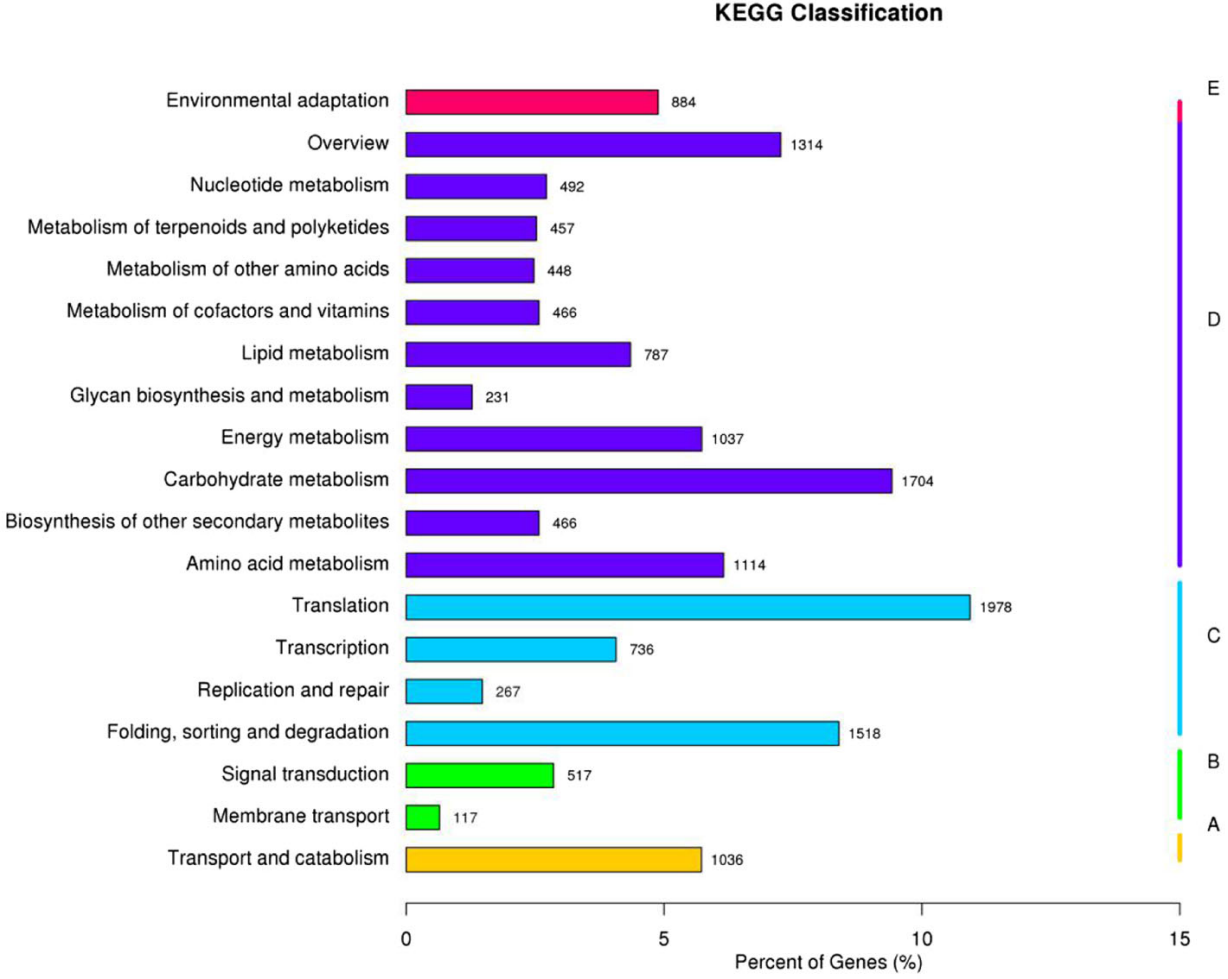
Histogram of cluster of KEGG pathways of assembled unigenes in *P. frutescens* seed. The horizontal axis is the gene number; and vertical axis is the name of cluster of KEGG. A: Cellular processes; B: Environmental information processing; C: Genetic information processing; D: Metabolism; and E: Organismal systems

To identify the biological pathways that were active in oil accumulation processes, all annotated unigenes were mapped to the reference pathways in Kyoto Encyclopedia Genes and Genomes (KEGG). In total, 15,569 unigenes were assigned to 5 clusters and 130 KEGG pathways (Fig. 4), including metabolism (amino acid metabolism, 1114 unigenes; biosynthesis of other secondary metabolites, 466; carbohydrate metabolism, 1704 unigenes; energy metabolism, 1037 unigenes; glycan biosynthesis and metabolism, 231 unigenes; lipid metabolism, 787 unigenes; metabolism of cofactors and vitamins, 466 unigenes; metabolism of other amino acids, 448 unigenes; metabolism of terpenoids and polyketides, 457 unigenes, nucleotide metabolism, 492 unigenes), genetic information processing (folding, sorting and degradation, 1518 unigenes; replication and repair, 267 unigenes; transcription, 736 unigenes, translation, 1978 unigenes), environmental information processing (membrane transport, 117 unigenes; signal transduction, 517 unigenes), cellular processes (transport and catabolism, 1036 unigenes) and organismal systems (environmental adaptation, 884 unigenes).

Interestingly, some pathways are closely related to variations in oil content, such as fatty acid biosynthesis (97 unigenes), biosynthesis of unsaturated fatty acids (78 unigenes), α-linolenic acid metabolism (76 unigenes), fatty acid elongation (160 unigenes), glycerolipid metabolism (39 unigenes) and glycerophospholipid metabolism (56 unigenes). These unigenes will provide critical clues to identify and characterize key genes involving in UFA and TAG biosynthesis in *P. frutescens* seeds.

### Analyses of differentially expressed genes at three seed development stages

Following the transcriptome assembly and annotation, clean reads obtained from each seed developing stage were individually mapped to determine the expression abundance as FPKM. Plotting expression fold change showed a high correlation of two biologically replicated sequencing runs as indicated by Pearson correlation (Additional file 4: Figure S2).

A total of 53 (32 up, 22 down), 653 (259 up, 394 down) and 1459 (742 up, 717 down) genes were differentially expressed in 10 DAF vs 5 DAF, 20 DAF vs 10 DAF, 20 DAF vs 5 DAF paired comparisons, respectively (log_2_ ratio ≥ 1 or ≤ − 1, FDR < 0.05) (Fig. 1c-f). Among all differentially expressed genes (DEGs), with the developmental process going on, the number of up-regulated genes was more than that of down-regulated genes. Differentially expressed genes will provide crucial cues to investigate the molecular mechanism of fatty acid synthesis and accumulation.

**Fig. 5:**
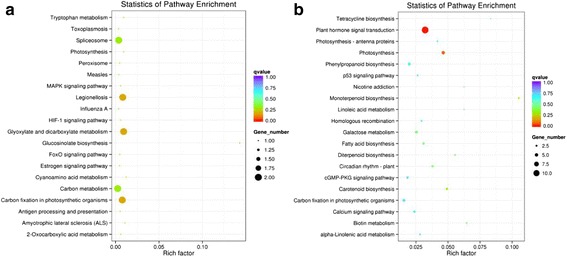
Scatterplot of KEGG pathway enrichment analysis of differential expressed genes in paired comparisons of 10 DAF vs 5 DAF and 20 DAF vs 10 DAF. **a** Gene numbers enriched in the pathways are less than 2.0. **b** Gene numbers enriched in the pathways are more than 2.5

To evaluate the potential functions of differentially expressed genes between two developmental stages, annotated genes were assigned to GO categories. In 10 DAF and 20 DAF seeds, functional categories linked to various metabolisms (organic substance, macromolecules, nitrogen compound), biosynthesis and oxidation-reduction, were highly enriched compared to 5 DAF seeds. To further understand their biological functions, they were mapped to KEGG pathways. In 10 DAF seed, pathways involved in glucosinolate biosynthesis, glyoxylate and dicarboxylate metabolism, carbon fixation in photosynthetic organisms, cyanoamino acid metabolism, spliceosome, tryptophan metabolism and photosynthesis were enriched (Fig. 5a, Additional file 5: Table S3). In 20 DAF seeds, enriched pathways closely related to fatty acid biosynthesis, linolenic and *α*-linoleic acid metabolism may provide valuable genetic resources for further characterization of FA accumulation during seed development (Fig. 5b, Additional file 6: Table S4).

### qRT-PCR validation

**Fig. 6:**
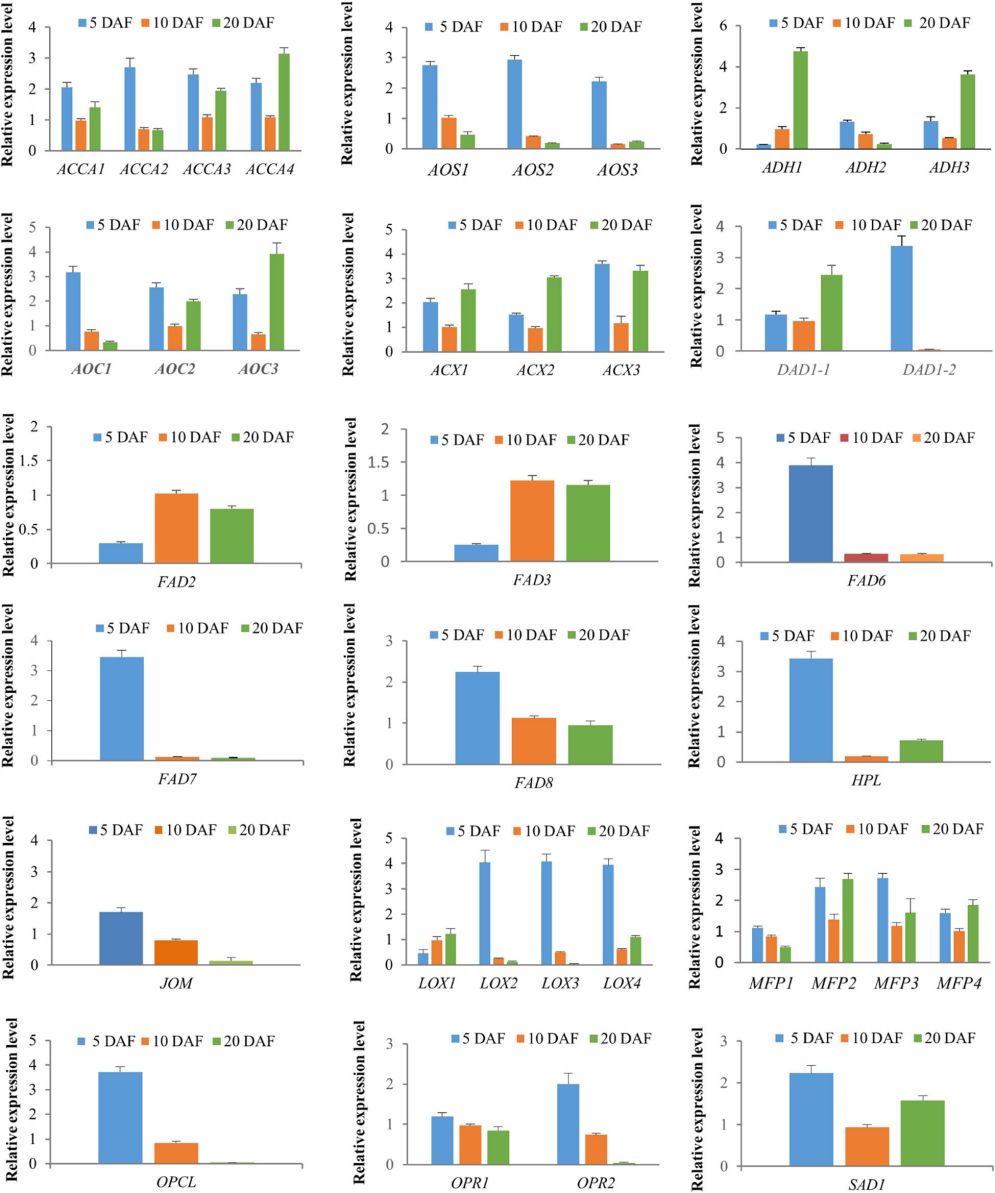
qRT-PCR validation of selected genes. The relative expression levels of unigenes were normalized with internal reference gene actin and 18sRNA. Values are means±SE with three replicated for each samples in qRT-PCR

To validate the reliability of RNA-seq results, 37 genes related to fatty acid biosynthesis and ALA metabolic pathway were selected for qRT-PCR validation. In general, qRT-PCR results of all genes except FAD6 displayed a high degree of consistency with RNA-seq results (Fig. 6). It was acceptable and rational that some differences in direct comparison between qRT-PCR and RNA-seq results would occur due to bias in library preparation for RNA-seq, different normalization approaches, and other technical biases [17, 18].

### Identification and expression profiling of fatty acid biosynthesis genes

**Fig. 7:**
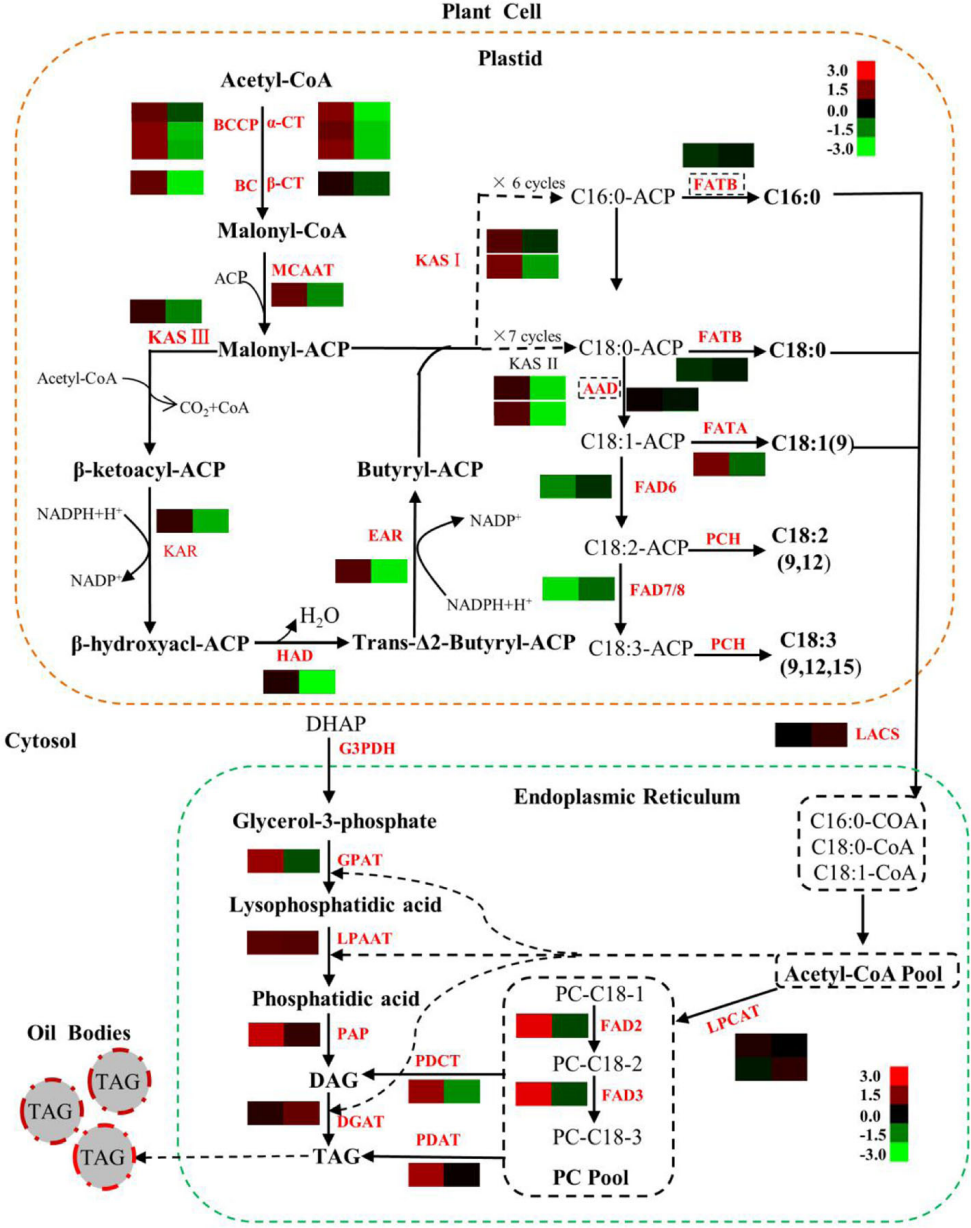
The reconstructed pathways of fatty acids biosynthesis in plastid and triacylglycerol biosynthesis in ER based on the de novo assembly and annotation of *P. frutescens* transcriptome. The icons besides the key enzymes represent the relative expression levels of their transcripts in seeds between 10DAF and 5DAF, 20DAF and 10DAF. The identified enzymes involved in fatty acid biosynthesis include α-CT, carboxyl transferase α-subunit; β-CT, carboxyl transferase β-subunit; BC, biotin carboxylase; BCCP, biotin carboxyl carrier protein; MCMT, malonyl-CoA ACP transacylase; KAS, ketoacyl-ACP synthase; KAR, ketoacyl-ACP reductase; HAD, hydroxyacyl-ACP dehydrase; EAR, enoyl-ACP reductase; SAD, stearoyl-ACP desaturase; FATA/B, acyl-ACP thioesterase A/B; FAD6, oleate desaturase (chloroplast-type); FAD7/8, linoleate desaturase (chloroplast-type). Enzymes involved in triacylglycerol synthesis are LPCAT: lysophosphatidylcholine acyltransferase; FAD2, oleate desaturase; FAD3, linoleate desaturase; GPAT, glycerol-3-phosphate acyltransferase (EC2.3.1.15);PAP, PA phosphatase (EC: 3.1.3.4); DGAT, acyl-CoA: diacylglycerol acyltransferase; PDAT, phospholipid:diacylglycerol acyltransferase (EC: 2.3.1.20); PDCT, phosphatidylcholine: diacylglycerol cholinephosphotransferase (EC:2.7.8.*)

Based on the KEGG pathway assignment, 35 unigenes encoding key enzymes involving in FA biosynthesis were successfully identified and their expression levels were compared between three developmental stages (Fig. 7). The biosynthesis pathway of FA was constructed by referencing previous reports [19, 20]. In plants, the de novo synthesis of fatty acid occurs primarily in the plastid and starts with the conversion of acetyl-CoA to malonyl-CoA, catalyzed by the rare-limiting enzyme acetyl-CoA carboxylase (ACCase; EC6.4.1.2) [19]. Heteromeric ACCase consists of four subunits: biotin carboxylase (BC), carboxyl transferase (α-CT and β-CT) and biotin carboxyl carrier protein (BCCP). All subunits except *β-CT* were significantly up-regulated in developing seeds from 5 DAF to 10 DAF, but down-regulated from 10 DAF to 20 DAF. Especially, the transcript of one α*-CT* homologous (c47294_g2) was increased 36-fold in 10 DAF seed (Additional file 7: Table S5). The expression level of *β-CT* was unchanged during the whole developmental process. As reported previously, the upregulation of ACCase could significantly alter the fatty acid composition and increased the FA contents in seeds, which further leaded to an increased oleic acid content [21, 22]. Thus, the overexpression of ACCase subunits in *P. frutescens* seed would contribute the accumulation of more substrates for the synthesis of FA from 5 DAF to 10 DAF. Subsequently, malonyl-CoA was transferred to the malonyl group by malonyl-CoA ACP transacylase (MCAAT, EC2.3.1.39) through an acyl carrier protein (ACP) to produce a malonyl-ACP, which is the primary substrate for the subsequent elongation. Next, series of condensation reactions were catalyzed by β-ketoacyl-ACP (KAS-III, EC2.3.1.180), NADPH-dependent β-ketoacyl-ACP reductase (KAR, EC1.1.1.100), dehydrated by 3-hydroxylacyl- ACP dehydratase (HAD, EC4.2.1.-) and enoyl-ACP reductase (EAR, EC1.3.1.9) to form C16:0-ACP or C18:0-ACP, which can further be catalyzed by stearoyl-ACP desaturase (SAD, EC1.14.19.2) to generate C16:1-ACP or C18:1-ACP [23]. In general, all these genes showed a bell shaped expression pattern, suggesting oil accumulation in *P. frutescens* seeds occurred after 10 DAF.

Finally, fatty acid synthesis is terminated by fatty acyl-ACP thioesterase (FATA, EC3.1.2.14) and palmitoyl/stearoyl-acyl carrier protein thioesterase (FATB, EC3.1.2.14) through moving the acyl group from ACP [24] . Interestingly, the expression of *FATA* (responsible for unsaturated FA production) was significantly increased from 5 DAF to 10 DAF, while the expression variation of *FATB* (for saturated FA production) was not observed during seed development. This was consist with higher plastid production of unsaturated than saturated FA in *P. frutescens* seed. In addition, one unigene encoding ω-6 fatty acid desaturase (FAD6, chloroplastic type), which catalyzes the formation of linolenic acid (C18:2), and one unigene encoding ω-7/8 fatty acid desaturase (FAD7/8, chloroplastic type), which catalyzes the formation of α-linolenic acid (C18:3), were identified and both of them exhibited an up-down-up expression pattern from 5 DAF to 20 DAF. Our results clearly indicated that linolenic acid and α-linolenic acid started to accumulate from 10 days after flowering, which was consistent with the lipid content measurement. Our results were also in agree with previous studies that most of the genes involving in the core fatty acid biosynthesis shared a similar temporal transcription pattern [20], suggesting the possibility that they were co-regulated and responsible for the higher oil accumulation in seeds. Free FA generated in the plastid were esterified to COA for triacylglycerol (TAG) biosynthesis by long-chain acyl-COA synthesis (LACs) at the plastid envelop. The expression of LACs peaked at 10 DAF suggested an increase in the flow of C16:0-COA, C18:0 COA or C18:1 COA towards TAG biosynthesis.

### Identification and expression profiling of transcripts involved involved in TAG biosynthesis and metabolism

The biosynthesis of TAG starts with the acyl transfer from acyl-CoA to glycerol-3-phosphate (G3P) to generate lysophosphatidic acid (LPA) catalyzed by glycerol-3-phosphate acyltransferase (GPAT). Subsequently, LPA is dephosphorylated by lysophosphatidic acid acyltransferase (LPAT) and phophatidate phosphatase (PAP) to produce sn-1,2-diacylglycerol [25, 26]. In *P. frutescens* seeds, unigene *GPAT*, *LPAT*, and *PAP* have a temporal expression pattern of “down-up-down”, which was consistent with the expression pattern of genes involved in FA biosynthesis. The biosynthesis of TAG is terminated by diacylglycerol acyltransferase (DGAT), a rate-limiting enzyme in the Kennedy pathway, which transfers an acyl group from acyl-CoA to sn-3 of DAG. The transcription level of *DGAT* kept constantly from 5 DAF to 10 DAF, however it was significantly increased at 20 DAF (Additional file 7: Table S5). This result indicated that TAG assembly and accumulation through glycolysis pathway occurred in seed around 20 DAF. Previous studies also demonstrated that over expression of *DGAT* could improve the oil content in Arabidopsis, soybean and maize seeds. In an alternative metabolic pathway, DAG could be generated from the conversion of the lipid phosphatidylcholine (PC) to DAG, which catalyzed by phosphatidylcholine:diacylglycerol cholinephosphotransferase (PDCT) (EC: 2.7.8.*). The transcripts encoding of PDCT also showed a bell-shaped expression pattern. This expression pattern indicated that its potential role in providing DAG pool enriched with PUFA around 10 DAF, which was further incorporated into the accumulation of TAG by DAGT. Thus, any variation of PDCT probably affects directly or indirectly both the level of FA saturation and the assembly of TAG.

Though TAG biosynthesis was believed to occur mainly through the glycerol pathway as described above, an alternative pathway known as the acyl-CoA independent pathway has also been reported in some plants, in which phospholipid:diacylglycerol acyltransferase (PDAT) transfers the sn-2 acyl group from acyl-CoA to phospholipid and generates TAG. It is interesting to note that the expression level of *PDAT* at 10 DAF and 20 DAF was significantly higher than that in seed at 5 DAF (Fig. 7 and Additional file 7: Table S5). The different expression profile of *PDAT* and *DGAT* strongly suggested that the transcriptional regulation of genes in the reactions of TAG biosynthesis were under separated controls.

The oil accumulation is regulated by the dynamic balance between synthesis and breakdown of TAG. Catabolism of TAG is initialed by the action of triacylglycerol lipase (TAGL) that breakdowns the ester bonds and releasing free fatty acid [27]. TAGL (c37957_gi) showed reduced expression (fold change = − 1.7) in 20 DAF seed than in 10 DAF seed. TAG catabolism proceeds in an opposite direction to the synthesis. Therefore, the suppression of TAG degradation would increase the accumulation of lipid content. Overall, identification of key genes involved in TAG biosynthesis and metabolism in our seed transcriptome helps to improve fatty acid and oil content in *P. frutescens* seeds.

### Identification and expression profiling of α-linolenic acid metabolism and jasmonic acid biosynthesis genes

Although ALA is enriched in *P. frutescens* seed, the molecular mechanisms underlying the accumulation of in developing seed is still unclear. Our results revealed that levels of palmitic, oleic, and linoleic acid were higher in 5 DAF seeds, whereas oleic and linoleic acids levels were significantly decreased from 5 DAF to 25 DAF (Fig. 1b). Further analysis revealed that the accumulation of ALA was correlated with the expression of fatty acid desaturases 2 (*FAD2*) and *FAD3*. Previous studies have shown that microsomal FAD enzymes are the major contributors to seed ALA content in soybean [28] and Arabidopsis [29]. Variation in the content of these fatty acids in developing seeds might be a result of differential activity of one or more desaturase enzymes. Therefore, it would be essential to study the genes involved in ALA content of *P. frutescens* seed varieties with specific emphasis on desaturase genes, in terms of copy numbers, allelic combination and transcriptional regulations as well as post-translational or post-translational regulation.

**Fig. 8:**
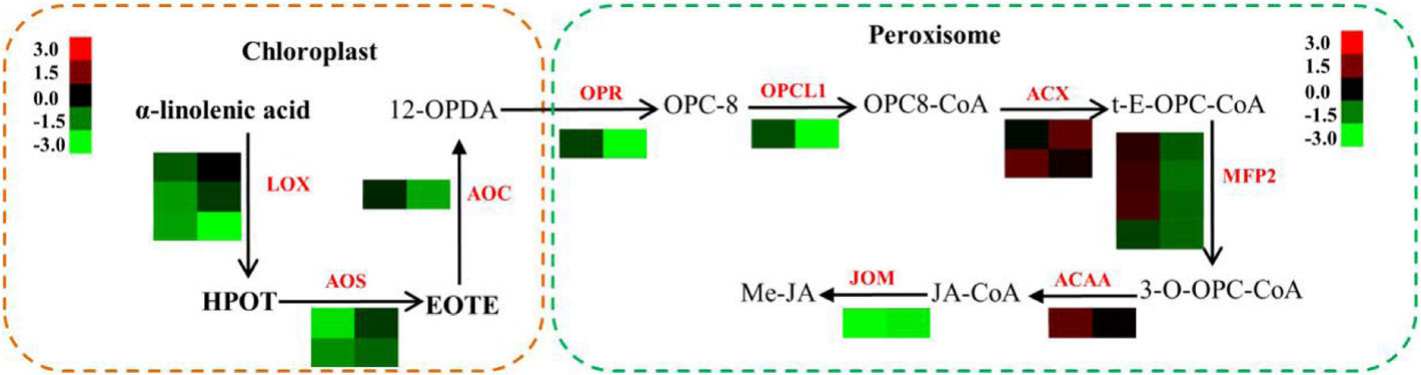
An integrated view of α-linolenic acid metabolism and in *P. frutescens*. HPOT, hydroperoxyoctadeca-9,11,15-trienoate; EOTE, 12,13-epoxyoctadeca- 9,11,15-trienoic acid; 12-OPDA, 12-oxophyto-10,15-dienoate; OPC-8, 8-[(1R,2R)-3-Oxo-2- {(Z)-pent-2-enyl} cyclopentyl]octanoate; 3-O-OPC-CoA, 3-Oxo-OPC8-CoA; t-E-OPC-CoA, trans-2-enoyl- OPC-8- CoA; JA-CoA, 7-isojasmonic acid CoA; Me-JA, methyl jasmonate; LOX, lipoxygenase; AOS, hydroperoxide dehydratase; AOC, allene oxide cyclase; OPR, 12-oxophytodienoic acid reductase; OPCL1, OPC-8:0 CoA ligase 1; ACX, acyl-CoA oxidase; MFP2, enoyl-CoA hydratase/3-hydroxyacyl-CoA dehydrogenase; ACAA, acetyl-CoA acyltransferase; JOM, jasmonate o-methyltransferase

Nine transcripts were identified and annotated as proteins involved in α-linolenic acid catabolism, including lipoxygenase genes (*LOX*), allene oxide synthase gene (*AOS*), allene oxide cyclase genes (*AOC*), OPDA(12-oxo-phytodienoic acid) reductase gene (*OPR*), OPC-8:0 (3-oxo-2((2Z)-pentenyl)- cyclopentane-1-octanoic acid) CoA ligase gene (*OPCL*), acyl-CoA oxidase gene (*AOX*), enoyl-CoA hydratase/3-hydroxyacyl-CoA dehydrogenase (*MFP2*),acetyl-CoA acyltransferase gene (*ACAA*) and jasmonate o-methyltransferase (*JOM*) (Fig. 8 and Additional file 7: Table S5). LOX and AOS are key enzymes regulating the ALA metabolism to generate other unsaturated fatty acids [30]. Our results showed that the expression level of *LOX* and *AOS* significantly decreased during seed development, which might benefit to the accumulation of ALA.

The biosynthesis of jasmonic acid in plant peroxisome requires the action of acyl-coenzyme A oxidase (ACX) through the octadecanoid pathway [31]. Briefly, α-linolenic acid is oxygenated by lipoxigenase (LOX), and then converted to 12-oxo-phytodienoic acid (12-oxo-PDA) by the sequential action of allene oxide synthase (AOS) and allene oxide cyclase. Subsequently, JA is further catabolized to generate its volatile counterpart MeJA [32]. Our results revealed that almost all genes encoding key enzymes involved in ALA metabolism in chloroplast were significantly down-regulated during the seed development process. There expression patterns extended the idea of suppressing ALA metabolism to increase its accumulation in mid-seed developmental stage.

### Identification of transcription factors involved in oil biosynthesis and deposition

**Fig. 9:**
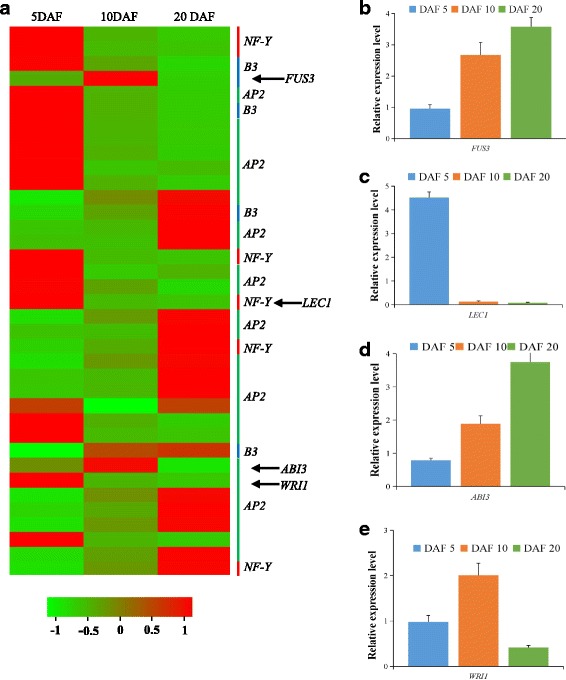
Expression profiles of differentially expressed members of transcription factor (TFs) family AP2, B3 and NFY putatively involved in oil biosynthesis and accumulation during seed development. **a**: Hierarchical cluster of expression levels of 37 TFs. Value of the color key refers to the log base 2 of gene expression level (RPKM).5 DAF (days after flowering), 10 DAF and 20 DAF were three stages of seed development. **b**-**e**: qRT-PCR validation of the expression of FUS3, LEC1, ABI3 and WRI1. The relative expression levels were normalized with internal reference gene Actin and 18 s RNA. Values are means ± SE with three replicated for each samples

Transcription factors (TFs) are important regulators which can modulate gene expression at the transcriptional level [33], but little is known about transcriptional regulation of lipid biosynthesis in *P. frutescens* seeds. This transcriptome analysis provides an opportunity to identify putative TF expression patterns during seed oil accumulation stages. Combined with the Plant Transcription Factor Database and literature comparison, 2769 TFs were identified, including oil store regulating genes WRINKLED1 (WRI1), LEAFY COTYLEDON1 (LEC1), FUSCA3 (FUS3) and Abscisic Acid Insensitive3 (ABI3), and GL2, and categorized into 81 families. The top five TF families were identified as C2H2 domain, MYB-related, HB, AP/EREB, and bHLH domain in terms of sequence abundance. Among these TF families, 37 transcripts were mapped to fatty acid and oil biosynthesis pathway, which belong to AP2, B3 and NFY families (Fig. 9a and Additional file 8: Table S7). The WRI1 protein, a member of Apetala2 ethylene response element binding factor (AP2/EREB), can up-regulate more than 10 genes in seed oil biosynthesis pathway [34, 35]. A transcription factor with high similarity to *WRI1* was significantly up-regulated in 10DAF seed when compared with 5DAF and 20 DAF seeds (Additional file 8: Table S7), During seed development, expression of several enzymes involved in FA biosynthesis pathways is regulated by the WRI1 transcription factor [36]. Over-expression of the WRI1 gene in *A.thaliana* and maize yield higher TAG content in vegetative tissues [37] and seeds. On the contrary, knockdown of *A. thaliana WRI1* could significantly reduce seed oil accumulation [36, 38]. Although, WRI appears to ubiquitously affect FA biosynthesis and TAG accumulation in diverse plants, its molecular regulatory mechanism of the specific lipid metabolism may differ in plant taxa. As an upstream transcription factors, LEC1 and LEC2 regulate the expressions of some genes putatively involved in key reactions of condensation, chain elongation, and desaturation of glycerolipid biosynthesis [39]. Seed-specific over-expression of maize *LEC1* (*ZmLEC1*) could increase ~ 48% oil accumulation in maize seed [40]. However, the expression of *LEC1* was decreased from 5DAF to 20DAF (Fig. 9a and Additional file 8: Table S7). One possibility is that it might be due to tissue- or species-specific regulatory differences in *LEC1* expression patterns. Of course, further studies will be needed to elucidate the regulatory mechanism of *LEC1* in oil accumulation in *P. frutescens* seeds. In addition, transcription factor ABI3 and FUS3 were well-represented in 10DAF seeds.

To validate the expression difference of identified TFs, about four TFs were selected for qRT-PCR analyses (Fig. 9b-e). The results of qRT-PCR confirmed that the transcriptome data. Because the main metabolic process in seeds is fatty acid and lipids synthesis, so the high expression level of these TFs might be involved in fatty acid and lipid synthesis. Of course, further studies will be performed to decipher their regulatory roles in oil synthesis. In summary, our results provide some valuable clues for understanding the molecular mechanism of fatty acid and lipids biosynthesis.

## Conclusions

Understanding the basic molecular mechanisms of lipid biosynthesis and metabolism is crucial for developing genetic engineering approaches for enhancing the oil content in *P. frutescens* seed. Detailed genome information is essential and indispensable for our understanding. Therefore, in this study RNA-seq data were generated from three developmental stages of *P. frutescens* seed. Transcripts encoding key enzymes involved in the biosynthesis and metabolism of fatty acids and TAG were successfully identified and pathways were reconstructed. These findings would provide useful information regarding the oil accumulation of *P. frutescens* seed.

Our results showed that the major time period for unsaturation fatty acids accumulation in *P. frutescens* seed under our experimental conditions occurred between 5 and 15 DAF. However, a significant portion of the transcriptome was highly dynamic between 10 DAF and 20 DAF. Gene ontology and KEGG pathway enrichment analysis revealed that biological processes such as FA and TAG biosynthesis, regulation of α-linolenic acid metabolism, were upregulated. Our de novo assembled transcriptome for *P. frutescens* seed will serve as an important resource for future genetic and evolutionary researches (comparison between varieties, and so on) that focus on gene expression differences at particular developmental period.

## Methods

### Plant materials

*Perilla frutescens var. crispa F. purpurea* (red *Perilla*) were grown in the experiment garden of Chongqing Normal University, China, under the natural conditions. Blooming plants were observed daily at the same time and tagged, and the tagging dates were recorded as 0 day after flowering (0 DAF). Seeds at 5 DAF, 15 DAF and 25 DAF were harvested for oil content measurements. Based on the results of FA composition test, seeds at 5DAF, 10DAF and 20 DAF were selected as materials for comparative transcriptome analysis to explore the regulation of fatty acid synthesis and metabolism. Two biological repeat samples were celected for each developmental stage, frozen immediately in liquid nitrogen and stored at − 80 °C until further use.

### Fatty acid composition determination

Seeds from each developmental stage were weighted and dried overnight at 60 °C, then were grounded, extracted with 1 mL of hexane for 1 h, centrifuged at 13,000 rpm for 10 min, and the upper suspensions were transferred to new tubes. This process was repeated for three times. To determine the profiles of fatty acids, extracted lipids were trans-methylated in 0.8 mL petroleum ether and 0.5 mL 5% H_2_SO_4_ (*v*/v, H_2_SO_4:_ methanol) and the resulting fatty acids were analyzed using GC-MS method (GC-2010, plus instrument, Shimadzu, Japan) with a flame ionization detector on a DB-23 column (60 mm × 0.32 mm ID× 0.25 μm df, Agilent Technologies, Waldbronn, Germany) with the following parameters: column oven temperature 170 °C and flame ionization detector set as 280 °C. Fatty acid content was expressed as percentage of total fatty acids.

### RNA extraction, library construction and sequencing

Total RNA was extracted from developing seeds at 5 DAF, 10 DAF and 20 DAF using TRIzol Reagent (Invitrogen, USA) with an additional DNAase I (QIAGEN) digestion step to remove any genomic DNA contamination according to the manufacturer’s directions. The purity and yield of total RNA was analyzed by the NanoDrop ND1000 spectrophotometer (Thermo Scientific, USA) and the Qubit Fluorimeter (Invitrogen, USA), respectively. The integrity was confirmed by the Aligent 2100 Bioanalyzer system (Agilent Technologies, USA). Samples with an RNA integrity number value greater than 8 were used for sequencing library preparation using Illumina®TruSeq™ RNA Sample Preparation Kit (Illumina Inc., San Diego, CA). Poly(A) mRNA was purified from 1 μg of total RNA using poly (T) oligo-attached magnetic beads according to the Illumina manufacturer’s instructions, and then fragmented by the Fragmentation Kit (Ambion, USA). Using these short fragments as templates, the first-strand cDNA was synthesized using ProtoScript II Reverse Transcriptase (Gibco, Life Technologies, USA) and random hexamers as primers. This step was followed by the second-strand cDNA synthesis using NEB second strand synthesis reaction buffer, DNA polymerase I and RNase H, The double stranded cDNA were purified using AMPure XP beads (Beckman Coulter, USA) and subjected to end repair process, adenylation and then ligated to Illumina multiplex barcode adapters. The adapter ligated cDNA was purified using AMPure XP beads and subjected to18 cycles of PCR to enrich the adapter-ligated fragments, which were further purified using AMPure XP beads. Sequencing libraries were initially quantified with a Qubit Fluorimeter (Thermo Fisher Scientific, USA) and diluted to a concentration of 1.5 ng/μL. The insert sizes were assessed by the Aligent 2100 Bioanalyzer system and then quantified by qPCR using the Kapa Library Quantification Kit (Kapa Biosystem, USA) (concentration > 2 nM). RNA sequencing was performed using a paired-end strategy (each end with 100 bases) on the Illumina HiSeq2000 platform at Biomaker Technology, Co., Ltd (Beijing China). The RNA-seq data was generated in FastQ format.

### Data processing, assembly and functional annotation

Raw reads were trimmed to obtain high-quality reads by removing the adaptor sequences, low-quality tags and ambiguous inner regions. Gene functions were annotated by homology searching against NCBI NR, SwissPort, and KOG databases using the BlastX with a cutoff of *E*-value ≤10^− 5^. Proteins with the best hits to the unigenes were used for functional annotations. Gene Ontology annotation was performed by the Blast2GO program [41] and classified by WEGO (http://wego.genomics.org.cn/cgi-bin/wego/index.pl). Unigenes were also aligned to COG database to predict and classify potential functions. To further annotate their possible metabolic pathways, unigenes were mapped to KEGG database by using the single directional best hit (SBH) method on the KEGG Automatic Annotation Server (KAAS) online (http://www.genome.jp/tools/kaas/) (*p* < 0.05).

### Transcription factor identification

To identify the transcription factors (TFs) represented in *Perilla* seed transcriptome, all assembled unigenes were searched against plant transcription factor database PlantTFDB (http://planttfdb.cbi.pku.edu.cn/) by BLAST with a cut-off of 1e^− 5^. Beased on the average of RPKM of genes across RNA-seq library replicates for a condition. Hierarchical clustering was performed using online Shinyheatmap server (http://shinyheatmap.com/). Before clustering, genes were filtered out that displayed low expression (< 5 RPKM) in all conditions. The average linkage method was used for cluster gerneration, with Euclidean distance as a similarity measure.

### Time-course differentially gene expression analysis

Gene set association analyses for two pairs of samples, 10 DAF vs 5 DAF, and 20 DAF and 10 DAF, were performed to identify genes/ pathways involved in fatty acid biosynthesis and metabolism significantly changed during seed development. The normalized RPKM (fragments per kb per million reads) was used to calculate the expression abundance of unigenes between samples. The *P*-values were adjusted for multiple comparisons by hypergeometric test / Fisher’s exact test and the Benjamini and Hochberg false discovery rate correction (FDR ≤ 0.05). An absolute value of ∣log_2_^ratio^∣ ≥ 1 was adopted as the cutoff to determine the significance of gene expression difference.

### Validation of differentially expressed genes by qRT-PCR

qRT-PCR was performed to validate the results of RNA-seq analysis, 18 differentially expressed candidate genes involved in FA biosynthesis and metabolism were selected. Specific primers were designed using Primer 5.0 and were listed in Additional file 9: Table S6. Total RNA were extracted from seeds using TRIzol Reagent and treated with DNase to remove genome contaminations. The first-strand cDNA was synthesized using a RevertAid First Strand cDNA Synthesis kit (Fermentas, Vilnius, Lithuania). qRT-PCR was performed using a SYBR kit (SYBR Green I, Osaka, Japan) on a LightCycler 480 system (Roche, Basel, Switzerland). The amplification conditions were as follows: 95 °C for 1 min followed by 40 cycles of 95 °C for 10 s, and 62–68 °C for 30 s (depending on different genes). Three independent biological triplicates and two technique repeats were performed for each sample. The relative gene expression levels were normalized to inner controls *β-actin* and 18*sRNA* and calculated using 2^-ΔΔT^ method [42] ANOVA analysis was performed using SPSS17.0 program (SPSS Inc., Chicago, USA). All data were represented as the means ± standard error (SE, *n* = 3).

## Additional files

Additional file 1: **Table S1.** Summary of sequencing and de novo assembly of *P. frutescens* seed transcriptome. (XLS 21 kb)

Additional file 2: **Figure S1.** Gene assembly and functional annotation results of *P. frutescens* transcriptome. A: Length distributions; B: Annotation statistic of seven databases; C:Venn diagram of gene annotations via selected five database searching; D:Species distribution of BLAST hits against NR protein database; E: E-value distribution of the best hits against the NR protein database; F:similarity distribution of the best hits against the NR protein database. (PNG 417 kb)

Additional file 3: **Table S2.** Summary of annotations for assembled unigenes in *P. frutescens* seed transcriptome. (XLS 21 kb)

Additional file 4: **Figure S2.** Correlation plot diagram of gene expression levels of two biological replicates. Horizontal axis and vertical axis refer the values calculated according to Log_10_^(FPKM + 1)^ of two replicates in each developmental stages (5 DAF, 10DAF and 20DAF). (PNG 273 kb)

Additional file 5: **Table S3.** Differentially expressed genes involved in fatty acid biosynthesis and metabolism. (XLS 26 kb)

Additional file 6: **Table S4.** KEGG pathway enrichment analysis of differentially expressed genes between 10 DAF and 5 DAF seeds. (XLS 58 kb)

Additional file 7: **Table S5.** KEGG pathway enrichment analysis of differentially expressed genes between 20 DAF and 10 DAF seeds. (XLS 34 kb)

Additional file 8: **Table S7.** List of differentially expressed TFs involved in fatty acid and TAG biosynthesis in *Perilla* seed. (XLS 13 kb)

Additional file 9: **Table S6.** Gene-specific primers used in qRT-PCR. (XLS 31 kb)

## Abbreviations

ABI3: Abscisic Acid Insensitive3
ALA: α-linolenic acid
DAF: days after flowering
FA: Fatty acids
FAD: Fatty acid desaturase
FUS3: FUSCA3
KEGG: Kyoto Encyclopedia of Genes and Genomes)
KOG: Eukaryotic orthologous groups
LEC1: LEAFY COTYLEDON1
SAD: Stearoyl-ACP desaturase
TAG: Triacylglycerol
WRI1: WRINKLED1
