# Supplementary figures and images for: Transcriptomic analysis of Perilla frutescens seed to insight into the biosynthesis and metabolic of unsaturated fatty acids
Source: BMC Genomics. 2018 Mar 21;19:213. doi: 10.1186/s12864-018-4595-z (PMC5863459; doi:10.1186/s12864-018-4595-z)

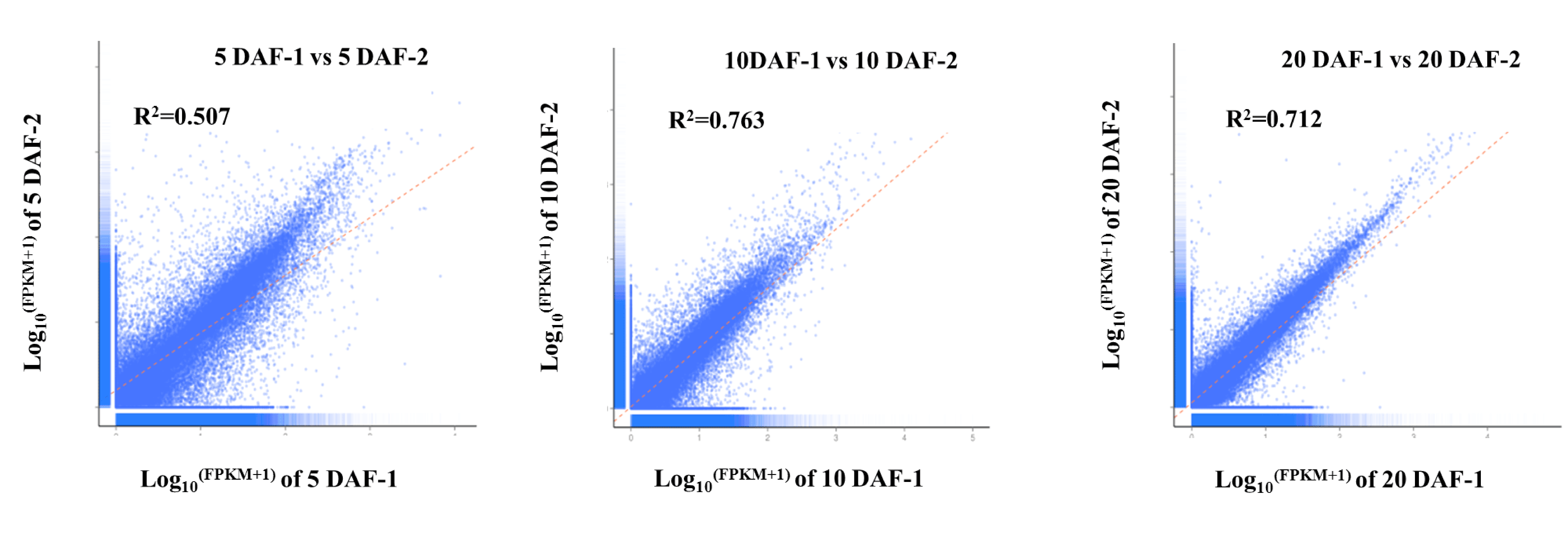

Supplement: Supplementary file 4 — Figure S2. Correlation plot diagram of gene expression levels of two biological replicates. Horizontal axis and vertical axis refer the values calculated according to Log10(FPKM + 1) of two replicates in each developmental stages (5 DAF, 10DAF and 20DAF). (PNG 273 kb) [file 12864_2018_4595_MOESM4_ESM.png]
